# Brown seaweed hydrolysate as a promising growth substrate for biomass and lipid synthesis of the yeast *yarrowia lipolytica*

**DOI:** 10.3389/fbioe.2022.944228

**Published:** 2022-08-17

**Authors:** Adam Dobrowolski, Willem Nawijn, Aleksandra M. Mirończuk

**Affiliations:** Department of Biotechnology and Food Microbiology, Faculty of Biotechnology and Food Science, Wroclaw University of Environmental and Life Sciences, Wroclaw, Poland

**Keywords:** brown seaweeds, hydrolysate, biomass, lipids, yarrowia lipolytica

## Abstract

Biomass of the brown algae *Fucus vesiculosus* and *Saccharina latissima* is a promising, renewable feedstock because of the high growth rate, accessibility and content of glucose and mannitol. Saccharification of seaweeds is a simple process due to the lack of lignocellulose in the cell wall. The high content of glucose and mannitol makes these seaweeds an attractive feedstock for lipid production in the yeast *Yarrowia lipolytica.* This study demonstrated that hydrolysates of brown algae biomass can be applied as a substrate for synthesis of yeast biomass and lipids without any supplementation. To increase the lipid titer in yeast biomass, we employed an engineered strain of *Y. lipolytica* overexpressing DGA1/DGA2. In consequence, the C/N ratio has a lower impact on lipid synthesis. Moreover, the applied substrates allowed for high synthesis of unsaturated fatty acids (UFA); the level exceeded 90% in the fatty acid pool. Oleic (C18:1) and linoleic acids (C18:2) achieved the highest content. The study showed that *Y. lipolytica* is able to grow on the seaweed hydrolysate and produces a high content of UFA in the biomass.

## Introduction

The growing global population requires an increasing amount of energy. It was predicted that by 2040, the global demand for energy will rise by 30% (Sönnischsen, 2020). Currently, fossil fuels remain the most common energy source, but due to the decline of these reserves, alternative feedstocks are being investigated, including biomass. The first generation of biomass consisted of crop plants, but there is a moral dilemma with using food for energy production. The second generation is lignocellulosic biomass, which before saccharification and fermentation requires costly pretreatment. The third-generation biomass is represented by algae and single cell oil (SCO) (Lee and Lee, 2016). This latter type of biodiesel possesses an advantage in the possibility to design the fatty acid profile. Depending on the aim of the application, the composition of the biofuel can be modified (Qiao et al., 2015). One of the bottlenecks in the biofuel industry is the economic feasibility of biofuel production, because the cost of the substrate might be a limiting factor. In the process of biodiesel production more than 90% of the operating cost comes from raw materials, of which almost 78% of the cost comes from feedstocks (Naveenkumar and Baskar, 2021). For this reason, several investigations to find a low-cost alternative feedstock for SCO production have been conducted (Papanikolaou and Aggelis, 2002; Papanikolaou et al., 2003; Tchakouteu et al., 2015; Sarris et al., 2017; Gottardi et al., 2021). Macroalgae are common in water ecosystems and, notably, they can grow at rates that far exceed those of terrestrial plants (Takagi et al., 2016). Brown algae are large, high growth organisms which do not require arable land, fertilizer or fresh water. Brown algae farms are popular in Asia and recently the production of macroalgal farms in the Baltic Sea was proposed (Kotta et al., 2022). The cell wall of the brown macroalgae is devoid of lignin, and thus the saccharification process is efficient. All this makes them a suitable, ethical feedstock for biomass production. Moreover, the biomass of macroalgae is rich in D-mannitol, a polyol that can be utilized by some microorganisms, such as *Yarrowia lipolytica* (Szczepańczyk et al., 2021). This yeast is known for its utilization of untypical carbon sources and production of high levels of lipid (SCO) under nitrogen limitation conditions from various types of waste products (Dobrowolski et al., 2019; Lopes et al., 2022). In *Y. lipolytica*, synthesis of triacylglycerol (TAG) occurs via the Kennedy pathway (Kennedy, 1962) and is widely used as a microbial cell factory due to the wide range of metabolic engineering tools, robustness, high tolerance for pH range and GRAS status (Mironczuk et al., 2018; Dobrowolski and Mironczuk, 2020; Wang et al., 2021). Previous research showed that genetic modification or applied substrates have an influence on the fatty acid profile (Papanikolaou et al., 2003; Qiao et al., 2015; Dobrowolski et al., 2016; Silverman et al., 2016), and might be modified depending on the purpose of the application. To increase the lipid content in yeast biomass, numerous studies have been conducted (Blazeck et al., 2014; Bellou et al., 2016), both to optimize the fermentation process (Papanikolaou et al., 2009) and by metabolic engineering (Abdel-Mawgoud et al., 2018). The genes involved in fatty acid synthesis in *Y. lipolytica* are the diacylglycerol (DAG) acyltransferase DGA1 gene (*YALI0E32769g*) (Athenstaedt, 2011) and the DGA2 (*YALI0D07986g*) (Beopoulos et al., 2012) gene, which requires acyl-CoA as an acyl donor. Their overexpression leads to enormous fatty acid accumulation in the biomass (Tai and Stephanopoulos, 2013; Friedlander et al., 2016); therefore, for this reason, a *Y. lipolytica* strain overexpressing DGA1/DGA2 was used in this study. The wild-type strain A101 (Wojtatowicz and Rymowicz, 1991) was used as a control.

While brown algae are potentially an abundant and suitable choice to use in a bioconversion process, to date they have not been used as part of a fermentation for SCO production. Moreover, while *Y. lipolytica* is an outstanding platform for SCO production, it was unknown whether brown algae-derived media will promote SCO production. The aim of this study was to investigate the possibilities to apply seaweed biomass as a substrate for biomass and fatty acid production by *Y. lipolytica*.

## Material and methods

### Strains and substrates

The strains used in this study were the wild-type A101 (Wojtatowicz and Rymowicz, 1991) from the collection of the Department of Biotechnology and Food Microbiology of Wrocław University of Environmental and Life Sciences, and its derived AJD DGA1/DGA2 overexpressing DGA1 and DGA2 genes. The following dried seaweeds were purchased: *F. vesiculosus* from Natvita and Flos (France), *S. latissima* from Seaweed Energy Solutions AS (Norway). These two seaweeds are natural and a common large alga on the shores of the Europe and a contain a high content of sugars in the biomass.

### Media and culture conditions

Rich Yeast Extract Peptone Glucose (YPD) medium was used for the yeast inoculum preparation and contained (g/L): 10 yeast extract (Merck, Germany), 10 peptone (Biocorp, Poland) and 20 glucose (Merck, Germany).

### Construction of AJD DGA1/DGA2 strain

First, an auxotrophy (*ura*) of strain AJD DGA1 (Dobrowolski et al., 2019) was restored *via* excision using the Cre-lox recombinase system as described before (Mirończuk et al., 2015). Next, the gene coding DGA2 (YALI0D07986g) was amplified from *Y. lipolytica* genomic DNA with primers DGA2_AscI_F: TAT​GGC​GCG​CCA​TGG​AAG​TCC​GAC​GAC​GAA and DGA2_NheI_R: TGC​GCT​AGC​TAA​AGG​CTA​CTG​ATG​AGT​G, resulting in a 1,625 bp PCR fragment. It was digested with the enzymes AscI and NheI and cloned into corresponding sites of the plasmid pAD (Mirończuk et al., 2017) under hybrid promoter UAS_B16-_TEF (Blazeck et al., 2013). The obtained construct pAD-DGA2 was sequenced (Genomed, Poland), digested with MssI, resulted in overexpression cassette surrounded with *Y. lipolytica* rDNA regions, and transformed to AJD pAD-DGA1 resulting in the strain AJD DGA1/DGA2. The obtained strain was confirmed for correct integration through gDNA extraction and three distinct PCR confirmations.

### Hydrolysis of seaweed biomass

First, the dry seaweed biomass was milled. For acidic hydrolysis 10% (w/v) seaweed biomass in water was treated with 0.2 M H_2_SO_4_ and autoclaved for 1 h (121°C). To neutralize the pH an appropriate amount of NaOH was added to pH 5. Enzymatic hydrolysis was performed by adding the Cellic CTec2 enzyme blend (4% v/v) (Novozymes, Denmark) to 10% seaweed biomass in 0.05 M citrate buffer with pH five at 50°C. The incubation took 24 h in incubation at rotary shaker 200 rpm. Next, the hydrolysates were spun down at 8,000 rpm for 10 min at RT, and the supernatants were filtered (MF-Millipore^®^ Membrane Filter, 0.45 µm pore size) and sterilized (autoclave, 121 C). Alginate was precipitated from the hydrolysates using 75 mm of CaCl_2_; subsequently, hydrolysates were spun down once more with aforementioned settings. 100 µL samples were 10x diluted in MQ water and run for HPLC analysis. The contents of mannitol and glucose were determined by HPLC using the Ultimate 3,000 system (Thermo Fisher) with a HyperREZ XP Carbohydrate H^+^ LC Column (Thermo Fisher) and an RI-101 detector (Shodex) connected to a desktop computer with Chromeleon software (Dionex) for data processing. The column was eluted with 25 mm of trifluoroacetic acid (TFA) at 65°C and a flow rate of 0.6 ml min^−1^. Subsequently, to optimize the enzymatic hydrolysis of brown algae biomass we tested: 1) concentration of enzyme dose (Cellic CTec2 enzymes from Novozymes) range 0–4% v/v; 2) optimal time of the process (24–72 h) and 3) optimal amount of the biomass, rage 10–20% w/v.

### Growth curves in spark microplates reader

The growth of the tested strains was measured using a Spark Microplate Reader (Tecan Group Ltd. Männedorf, Switzerland). First, the inoculum strains were grown for 24 h in YNB medium (Sigma-Merk, Germany) supplemented with 2% (w/v) glucose. Next, the cultures were spun down at 5,000 RPM for 3 min and washed twice with sterile Milli-Q water. The strain was grown on hydrolysates from *F. vesiculosus* and *S. latissima* seaweeds supplemented with YNB medium or (NH_4_)_2_SO_4_. Cultures were maintained at 28°C under constant agitation, the optical density of each culture was measured every 30 min for 96 h and each condition was performed with five replicates.

### Shake flask experiment: The time course of *Y. lipolytica* growth on seaweed hydrolysate

Strain A101 derivatives were grown in a 0.3 L flask in 50 ml of seaweed hydrolysate from *F. vesiculosus* and *S. latissima* at 28°C for 72 h*.* Samples were taken every 8 h, for OD measurement, the biomass collection and sugar consumption. OD600 was determined using a SmartSpec spectrophotometer (Bio-Rad). 10 ml of yeast culture was spun down, 5,000 RPM for 3 min, then the pellet was resuspended in 2–4 ml of demi water and filtered. The cells were then dried and weighed using a 50. R weighing dryer (RADWAG). The concentrations of mannitol and glucose were determined with HPLC as described above.

### Lipid extraction and fatty acid characterization

The fatty acids (FAs) from lyophilized biomass were derivatized to fatty acid methyl esters (FAMEs) using a method described before (Browse et al., 1986). Briefly, biomass (approximately 10–20 mg) was mixed with 2 ml of 2.5% sulfuric acid in methanol (containing 50 μg/ml of C17:0 as an internal standard) in glass tubes with Teflon caps, vigorously mixed for 2 min and incubated at 80 °C for 90 min to form FAMEs. FAMEs were extracted by adding 1 ml of hexane and 0.5 ml of water, mixed and spun down (centrifuged) for batter phase separation. The organic phase containing FAMEs was transferred into glass vials for GC analysis. FAMEs were analyzed by gas chromatography on GC-2010 Plus apparatus (Shimadzu, Japan) with a flame ionization detector (FID) and autoinjector (AOC-20i). The separation of FAMEs was achieved using a 70% cyanopropyl polysilphenylene-siloxane column (TR-FAME, 30 m × 0.32 m × 0.25 µm). The initial oven temperature was 130°C held for 1 min, which was then increased to 200°C at the rate of 5°C × min^−1^, then increased to 250°C at rate of 10°C × min^−1^ and held for 1 min. Temperatures for the injector and detector were 270 and 280°C, respectively. Helium was used as the carrier gas with constant flow of 1.52 ml × min^−1^. Volume of injection was 1 µL with a split rate of 1:5.

The identification of FAME was evaluated using Supelco 37 Component Fame Mix as a reference standard and for quantification analysis heptanoic acid was used as an internal standard. The total lipid content in dry cell weight was calculated as the sum of all fatty acids.

## Results and discussion

### Optimization of seaweed biomass hydrolysis

**FIGURE 1:**
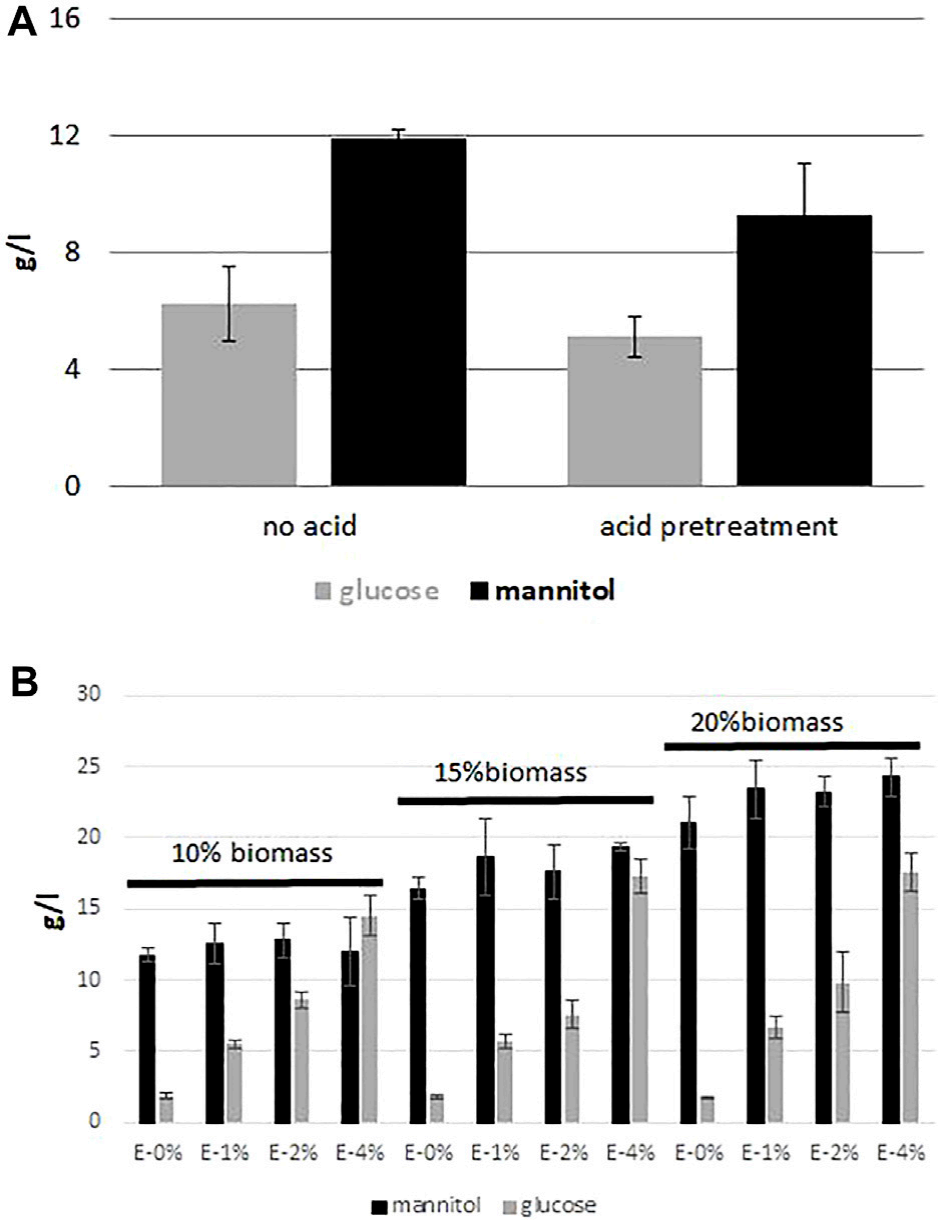
Effect of different treatment conditions on glucose and mannitol release from F vesiculosus biomass hydrolysate. **(A)** “acidic vs. no acidic” treatment of hydrolysate before enzymatic digestion, **(B)** effect of biomass load and enzyme Cellic CTec2 dosage.

Despite the fact that biomass of seaweed is rich in glucose and mannitol, a saccharification process is required to release them. Many studies have been conducted to optimize the hydrolysis process, both acid and enzymatic (Adams et al., 2008; Manns et al., 2016; Ravanal et al., 2016). First, in our study we focused on the acid hydrolysis of brown seaweed biomass and we addressed the question whether the acid pre-treatment is required for glucose and mannitol release from brown macroalgae biomass. In this step we employed *F. vesiculosus* biomass to compare two different conditions, with and without acid pre-treatment. The biomass was treated as described in Material and Methods, and the sample was analyzed by HPLC. As seen in Figure 1A, the differences between the samples with or without acid treatment are small. The level of glucose was 5.1 g/L and 6.2 g/L for biomass treated with acid and not treated with acid, respectively. The mannitol level was higher was for the sample without acid treatment and it was 11.9 g/L, whereas after acid treatment it was 9.3 g/L. It is important to note that acid hydrolysis is energy-consuming, since the hydrolysis takes 1 h at 120°C.

Next, we optimized the enzymatic hydrolysis of brown algae biomass by using a varying range of enzyme dose (Cellic CTec2 enzymes from Novozymes), testing the optimal time of the process and the amount of the treated biomass. In this experiment we tested four different concentrations of the enzymes (0–4% v/v) and three different concentrations of biomass, 10, 15 and 20% w/v. As seen in Figure 1B, the best results were observed for the samples with 4% of enzymes for all concentrations of biomass, where the amount of the released glucose was the highest and it ranged from 14.5 g/L to 17.5 g/L. In addition, concentration of mannitol ranged from 12.0 to 24.3 g/L. However, the relative viscosity of the samples containing 15 and 20% of seaweed biomass was very high (data not shown); thus for this reason the next experiments were continued with 10% biomass with 4% of the applied enzyme without acid-thermal hydrolysis.

### Seaweed as a feedstock for oleaginous yeast

**FIGURE 2:**
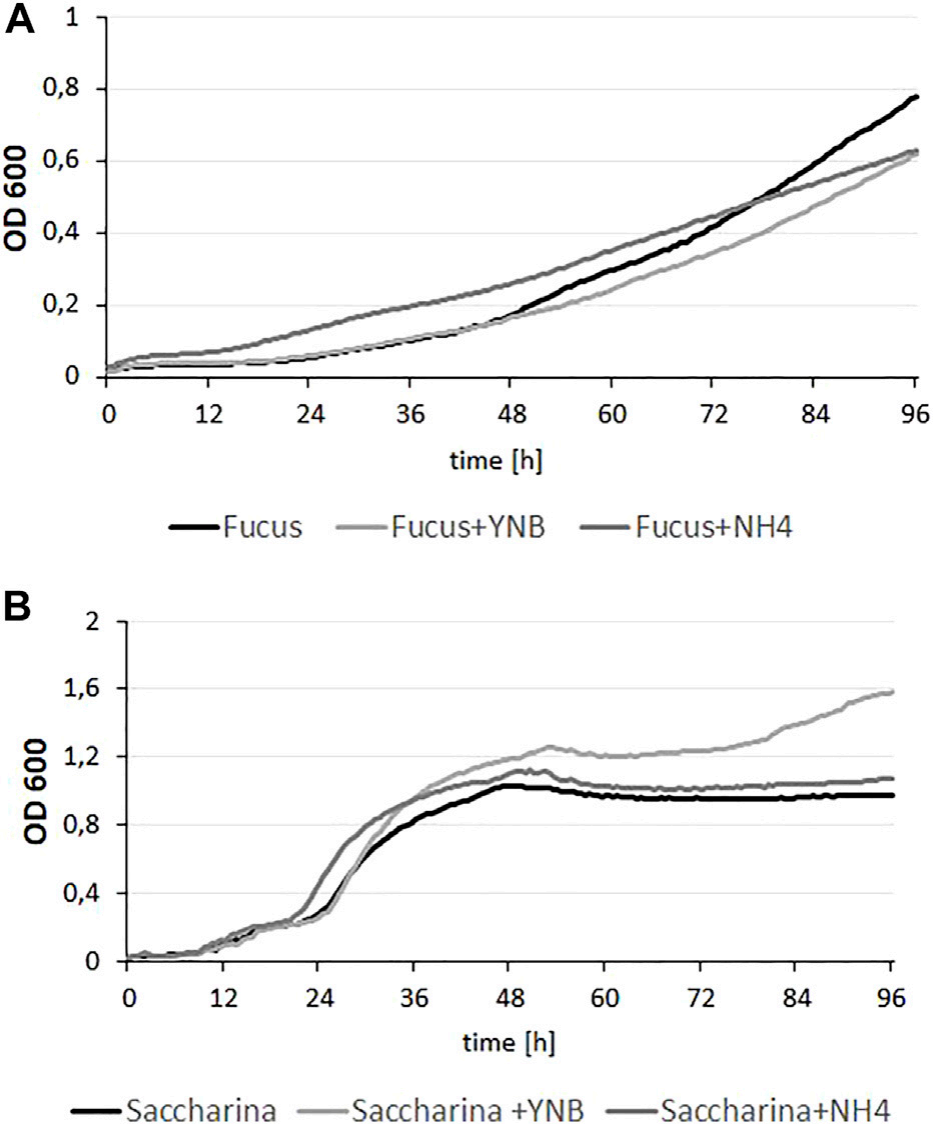
Growth curves of Y. lipolytica on hydrolysates from F. vesiculosus **(A)** and S. latissima **(B)** seaweeds, supplemented with YNB (6,8 g/L) medium or (NH_4_)_2_SO_4_ (5 g/L). Cultures were maintained at 28°C under constant agitation, optical density of each culture was measured every 30 min and each condition was performed with five replicates.

*Y. lipolytica* is known for its ability to utilize various types of substrates, including glycerol, alkanes and polyols (Ledesma-Amaro and Nicaud, 2016; Rzechonek et al., 2017). Despite this fact, its capacity to grow on brown algae hydrolysate has never been tested. Moreover, biomass of brown algae has been never described as a proper substrate for SCO synthesis. To date, seaweed biomass has been used as a substrate for mainly engineered microorganisms to produce lactic acid (Nagarajan et al., 2022), riboflavin (Perez-Garcia et al., 2022) or fucoxanthin (Pajot et al., 2022). Thus, to verify this we used a wild-type strain and a microplate experiment was performed. As substrates, two different types of brown algae hydrolysates of *F. vesiculosus* and *S. latissima* biomass were used. Because we did not test the level of nitrogen in the biomasses, first the hydrolysates were supplemented with YNB medium or (NH_4_)_2_SO_4_, As seen in Figure 2A, yeast growth on *F. vesiculosus* hydrolysate did not show a typical growth curve; the phase of logarithmic growth is strongly flattened. At the beginning the medium containing hydrolysate supplemented with (NH_4_)_2_SO_4_ seemed the most suitable for *Y. lipolytica* growth and supplementation of the hydrolysate with YNB did not have a positive effect on growth rate. However, at the end of the process, all growth curves reached a similar level, and any supplementation had a strong effect on growth. Probably, the poor growth rate was caused by a low concentration of dissolved oxygen (DO) in the medium. As was shown before, the yeast *Y. lipolytica* demands a high DO level (Mirończuk et al., 2019). The yeast growth on *S. latissima* hydrolysate showed an opposite effect. The growth rate was high for all conditions; the yeast achieved OD < 0.8 already after 24–36 h, and the highest OD (1.6) was observed for the strain growing on hydrolysate supplemented with YNB. For both hydrolysates, growth was observed, but for *S. latissima* the growth rate was higher. The content of glucose of both hydrolysates was at a similar level (around 15 g/L) but the content of mannitol was higher in biomass of *F. vesiculosus* (12 g/L). As mentioned above, probably the growth of yeast on the latter hydrolysate was limited by lack of DO caused by high viscosity (data not shown). Since the obtained results showed that hydrolysates are a suitable source for yeast growth, we performed an experiment on a larger scale.

### Biomass and lipid production by *Y. lipolytica* grown on hydrolysates

**FIGURE 3:**
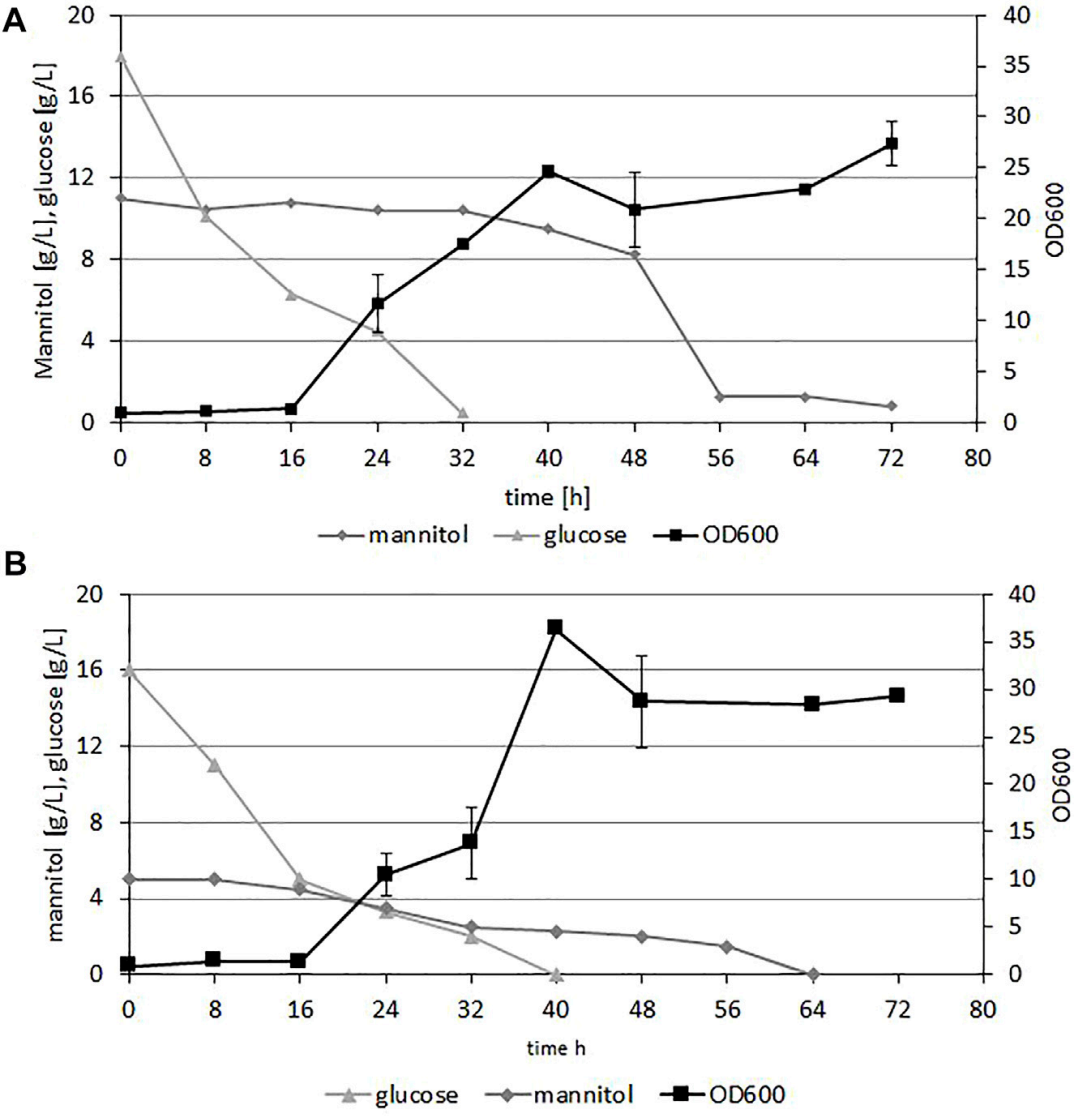
Time course of Y. lipolytica A101 growth (OD600) and fermentable sugar concentration during growth on F vesiculosus **(A)** and S. latissima **(B)** hydrolysates in shake flasks experiment. The strain was grown in 250 ml flasks at 28°C, with 200 rpm shaking in 50 ml medium. Error bars indicate SD from three biological repetitions.

To demonstrate the growth of *Y. lipolytica* on the hydrolysates from brown algae biomass we performed shake-flask experiments. As the microplate results showed small differences in growth rates between hydrolysates with or without supplementation with YNB or (NH_4_)_2_SO_4_, to reduce the medium cost we applied sole hydrolysate with 5% enzyme to increase the amount of the released glucose and mannitol. The OD was measured every 8 h for 72 h. Figure 3 shows the results of *Y. lipolytica* A101 growth on both types of hydrolysates and glucose and mannitol utilization. The initial concentration of glucose was 17 g/L and 18 g/L and mannitol 11.0 g/L and 3.0 g/L for *F. vesiculosus* and *S. latissima,* respectively*.* As a first carbon source glucose was utilized within 32–40 h. After depletion of this carbon source, utilization of mannitol started. This utilization was much more prolonged; it started slowly from the beginning of the fermentation, but took 64–72 h for both types of hydrolysates. Biomass production of the yeast was robust, and we observed all phases of growth on media based on both types of hydrolysates. The process took 72 h, and within this period the glucose and mannitol were utilized by the yeast. These data showed that hydrolysates from seaweed are a good substrate for production of yeast biomass. As mentioned above, brown algae is an abundant feedstock, is 33.6% of the globally farmed seaweed (Radulovich et al., 2015); moreover, in comparison to terrestrial plants, seaweeds have a higher growth rate and areal productivity. Seaweeds contain high carbohydrate concentrations of fermentable sugars, and because they can be cultivated in offshore or onshore systems in open seas, they do not interfere with food production (Takagi et al., 2016). Due to the promising results we employed the engineered strain AJD DGA1/DGA2 to produce elevated levels of fatty acids from the seaweed hydrolysates.

### Fatty acid profiles

The yeast *Y. lipolytica* possesses the capacity for accumulation of fatty acids in lipid bodies (Bellou et al., 2016), a process that occurs under nitrogen limitation conditions combined with excess of carbon (André et al., 2009). The optimal C/N ratio for lipid production ranges from 60 to 100, and the carbon source also has an influence on fatty acid production (Dobrowolski et al., 2016; Ledesma-Amaro et al., 2016). Since the C/N ratio in the brown algae may vary between species and it depends on the season (Chen et al., 2022), here we did not optimize the C/N ratio for fatty acid production. To enhance the level of fatty acids in the yeast biomass we employed DGA1 and DGA2 overexpressing strain. In a previous experiment we determined that the total available carbon in hydrolysates is utilized within 72 h; thus the samples for the biomass and fatty lipid profile were taken after this period.

**FIGURE 4:**
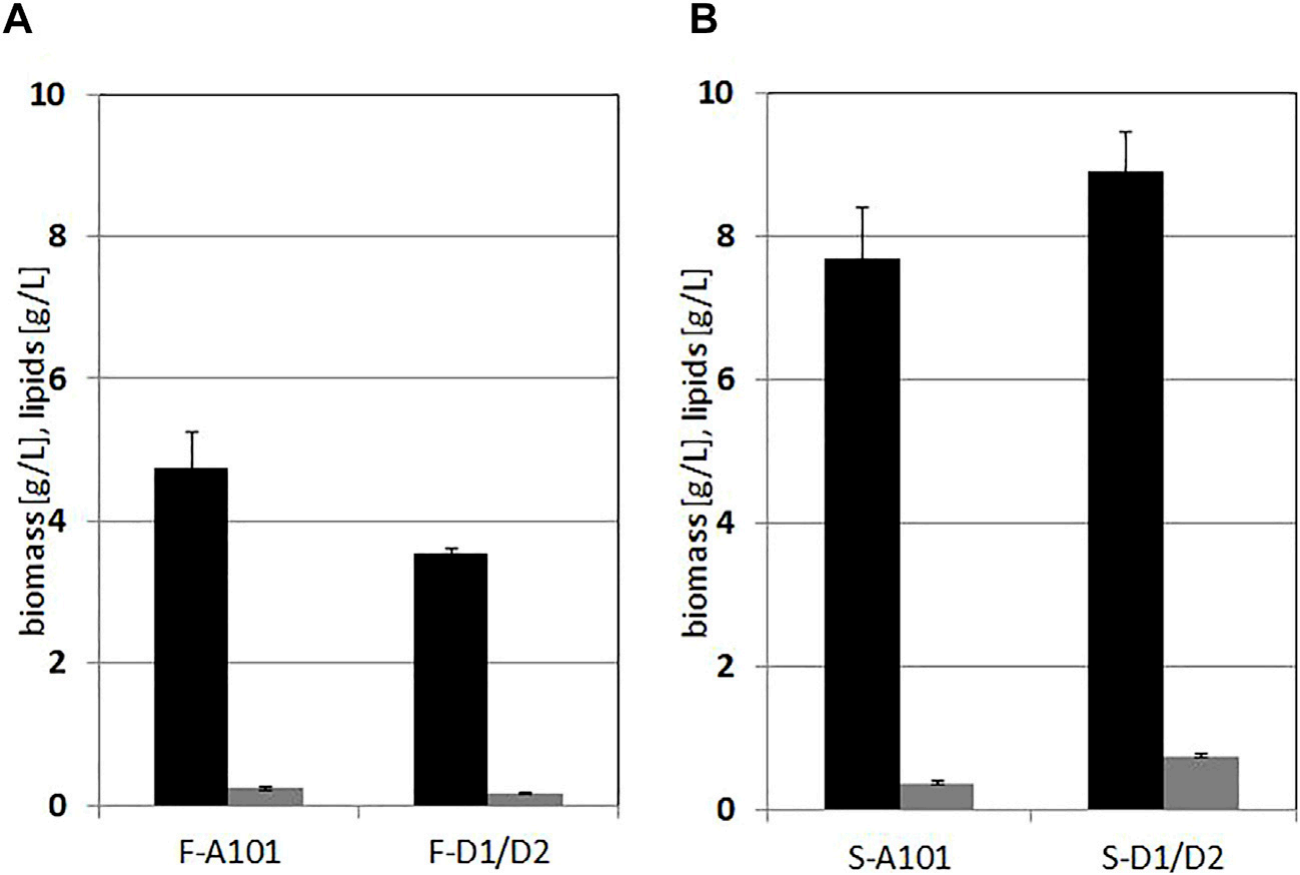
Lipid (grey bars) and biomass (black bars) production by Y. lipolytica A101 and AJD DGA1/DGA2 strains grown on F vesiculosus—**(A)** and S. latissima **(B)** hydrolysates. Cells were grown 72 h in 250 ml flasks at 28°C, with 200 rpm shaking in 50 ml medium. Error bars indicate SD from three biological repetitions.

Production of biomass for both strains on hydrolysates from *F. vesiculosus* was moderate and the lipid content in dry biomass was low. For the wild type, biomass of 4.75 g/L was achieved and for the engineered strain 3.55 g/L, and the lipid content was 5% (Figure 4). However, the hydrolysate based on *S. latissima* was a more suitable source for biomass and lipid production. The wild type biomass reached 7.7 g/L, the engineered strain 8.9 g/L. The content of fatty acids in the biomass was still moderate (below 10% for both strains) but elevated for the DGA1/DGA2 overexpressing strain. Additionally, strains growing on the hydrolysates from *F. vesiculosus* with the higher mannitol concentration had a lower SCO yield, which is a puzzling result. Most probably, it contains inhibitory compounds, which can also be an explanation for atypical growth (Figure 2A). However this hypothesis requires further study.

Most interesting were fatty acid profiles for both applied substrates. The unsaturated fatty acids constituted more than 90% in the fatty acid pool (Table 1). Most likely, this effect is caused by high content of unsaturated fatty acids in algae biomass (Wang et al., Forthcoming 2022). *De novo* synthesis of unsaturated fatty acids by *Y. lipolytica* from various substrates has been done before. However, the content in the total fatty acids pool did not exceed 60% (Kothri et al., 2020; Kieliszek and Dourou, 2021). In addition, supplementation of the synthetic medium with magnesium increased fatty acids content in the biomass, but did not improve the unsaturated fatty acids pool (Bellou et al., 2016). Despite this fact, *ex novo* synthesis of FA by *Y. lipolytica* allowed for high content of unsaturated fatty acids content, ranged from 70 to 90% (Lopes et al., 2018; Lopes et al., 2019; Pereira et al., 2022).

**TABLE 1:** Fatty acids profile of the strains A101 or AJD DGA1/DGA2 grown on *F. vesiculosus* (F) or *S. latissima* (S) hydrolysates.

Profile of fatty acid (%)									
	C16:0	C16:1	C18:0	C18:1	C18:2	Others	SFA	MUFA	PUFA
F-A101	5.90	8.45	0.94	43.49	34.48	6.74	6.83	51.94	34.48
F-D1/D2	6.79	5.46	0.96	63.68	19.05	4.05	7.76	69.14	19.05
S-A101	6.16	7.25	0.62	37.67	34.26	14.04	6.78	44.91	34.26
S-D1/D2	5.39	8.04	3.77	52.32	22.25	8.23	9.16	60.37	22.25

The major fatty acid detected in *Y. lipolytica* biomass was oleic acid (C18:1), and it ranged from 37.67 to 63.68% of the fatty acid pool. The second was linoleic (C18:2), which was accumulated from 19 to 34%. These results agree with previous reports (Makri et al., 2010; Poli et al., 2014), but in contrast, we observed a very low level of stearic acid (C 18:0): the concentration did not exceed 4% (0.62–3.77%). Interestingly, not all brown algae resulted in the same lipid profile, which suggests that a preliminary study should be done to select the appropriate seaweed for each SCO production process.

Yeast biomass rich in unsaturated fatty acids might be considered as a food supplement because PUFAs are important nutrients in the human diet. The positive influence of PUFAs on human health is known and well-studied (Tan et al., 2022). Moreover, anti-inflammatory effects were reported for oleic acid (Farag and Gad, 2022), which demonstrated a positive impact on human health.

The possibility of modifying the fatty acid profile in *Y. lipolytica* has been widely studied, and this goal can be achieved by genetic modification (Blazeck et al., 2014; Ledesma-Amaro et al., 2016; Abdel-Mawgoud et al., 2018) or by substrate selection and optimization of conditions (Papanikolaou and Aggelis, 2002; Papanikolaou et al., 2003; Dobrowolski et al., 2016; Mavrommati et al., 2022; Pereira et al., 2022). Depending on the purpose, the fatty acid profile can be switched to one with more saturated fatty acids, which is desired for biodiesel production, or unsaturated fatty acids, which is desired for food or feed supplements.

## Conclusion

This study showed that hydrolysate from macroalgae may be applied without any supplementation as a substrate for biomass and lipid production by *Y. lipolytica*. Moreover, acidic pretreatment of seaweed biomass is not necessary for efficient glucose and mannitol release. The wild type and the engineered strains grown on *S. latissimi* hydrolysate showed higher biomass and lipid production. The engineered strain AJD DGA1/DGA2 showed an elevated lipid content in the biomass. Despite the fact that lipid biosynthesis by *Y. lipolytica* on seaweed hydrolysates has to be optimized, the fatty acid composition of the oil produced by *Y. lipolytica* growing on this substrate supports its potential use as a feedstock.

## Data Availability

The raw data supporting the conclusions of this article will be made available by the authors, without undue reservation.
