# Supplementary material for: Brown seaweed hydrolysate as a promising growth substrate for biomass and lipid synthesis of the yeast yarrowia lipolytica
Source: Front Bioeng Biotechnol. 2022 Aug 17;10:944228. doi: 10.3389/fbioe.2022.944228 (PMC9428158; doi:10.3389/fbioe.2022.944228)
Supplement: Supplementary file 1 [file Table1.DOCX]

| **Profile of Fatty Acid (%)** | | | | | | | | | |
| --- | --- | --- | --- | --- | --- | --- | --- | --- | --- |
|  | **C16:0** | **C16:1** | **C18:0** | **C18:1** | **C18:2** | **others** | **SFA** | **MUFA** | **PUFA** |
| **F-A101** | 5,90 | 8,45 | 0,94 | 43,49 | 34,48 | 6,74 | 6,83 | 51,94 | 34,48 |
| **F-D1/D2** | 6,79 | 5,46 | 0,96 | 63,68 | 19,05 | 4,05 | 7,76 | 69,14 | 19,05 |
| **S-A101** | 6,16 | 7,25 | 0,62 | 37,67 | 34,26 | 14,04 | 6,78 | 44,91 | 34,26 |
| **S-D1/D2** | 5,39 | 8,04 | 3,77 | 52,32 | 22,25 | 8,23 | 9,16 | 60,37 | 22,25 |

Table 1. Fatty acids profile of the strains A101 or AJD DGA1/DGA2 grown on *F. vesiculosus* (F) or *S. latissima* (S) hydrolysates.
